# Dockres: a computer program that analyzes the output of virtual screening of small molecules

**DOI:** 10.1186/1751-0473-5-2

**Published:** 2010-01-14

**Authors:** Mihaly Mezei, Ming-Ming Zhou

**Affiliations:** 1 Department of Structural and Chemical Biology, Mount Sinai School of Medicine, One Gustave L Levy Place, New York, New York 10029, USA

## Abstract

**Background:**

This paper describes a computer program named Dockres that is designed to analyze and summarize results of virtual screening of small molecules. The program is supplemented with utilities that support the screening process. Foremost among these utilities are scripts that run the virtual screening of a chemical library on a large number of processors in parallel.

**Methods:**

Dockres and some of its supporting utilities are written Fortran-77; other utilities are written as C-shell scripts. They support the parallel execution of the screening. The current implementation of the program handles virtual screening with Autodock-3 and Autodock-4, but can be extended to work with the output of other programs.

**Results:**

Analysis of virtual screening by Dockres led to both active and selective lead compounds.

**Conclusions:**

Analysis of virtual screening was facilitated and enhanced by Dockres in both the authors' laboratories as well as laboratories elsewhere.

## Background

Virtual screening of small molecules is a widely used *in-silico *technique as an initial step towards development of selective chemical ligands that functionally modulate a target protein [1-3]. Such screening typically involves minimization of a scoring function calculated at atomic-scale resolution. Employing computationally efficient algorithms allows screening of a large chemical library. Since the 'scoring-function landscape' is highly nonlinear, that would there are require repetition of the minimization several hundred times. This means that a single screen of a chemical library will generate a large number of complexes - described as 'poses' - whose visual examination is almost impossible for all practical purposes. This note describes a computer program that is designed to sort and filter a large set of poses by various selection criteria and extract the filtered complexes.

## Methods

Dockres, written in Fortran-77, scans the output files of Autodock-3 or Autodock-4 [4] resulting from screening of a library of chemical ligands and extracts the docked poses and their calculated scores. From the coordinates of a macromolecule (target) the environment of the docked poses can be established.

There is an option to adjust the calculated free energy score with a contribution based on the multiplicity (*m*) of each pose [5]:(1)

**Figure 1:**
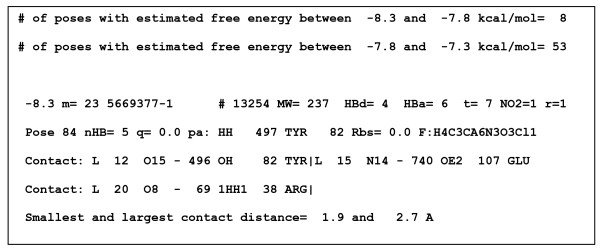
**Characterization of a docked pose of one ligand**. In the first line, the free energy score (-8.3) is specified, followed by it multiplicity (m), the ligand file name (5669377-1), the molecular weight (MW), the number of hydrogen-bond donors and acceptors (HBd and HBa), the number of torsions (t), -NO_2 _groups (NO2) and rings (r). The second line specifies the pose number (84), the number of hydrogen bonds between the ligand and the protein (nHB), the ligand charge (q), the protein atom (atom and residue names and numbers) closest to the ligand (pa), the distance from the binding site (Rbs) and the chemical formula (F). The subsequent lines describe ligand-protein contacts: ligand atom (L) number and name - protein atom number, name, residue number and name. Contact is defined as pairs of ligand-protein atoms that are mutually proximal. In addition, the pose list is preceded by statistics giving the number of poses in different free-energy score range.

The extracted poses are sorted by their scores calculated by Autodock. Figure 1 shows a typical record describing a pose (ligand properties, contacts, etc.). The user has an option of selecting the number of top scoring poses to list; another option is to limit each ligand to a single pose in this list. Besides the list of top-scoring poses and their characterizations, the docked complexes can be extracted into either a single PDB file with all the selected poses or separate PDB files, each containing a complex with a single ligand.

**Figure 2:**
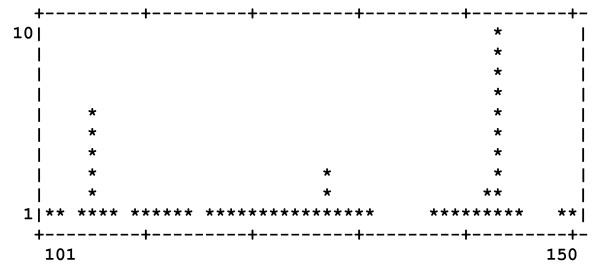
**Distribution of the number of poses closest to residues 101-150 of uPAR/α5β1**.

**Figure 3:**
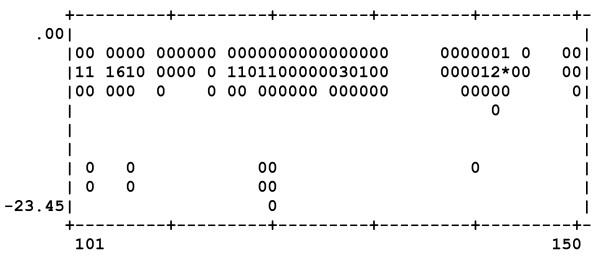
**Distribution of scores of the poses closest to residues 101-150 of uPAR/α5β1**. The symbol * represents the highest occurrence, the digits 0-9 give the occurrence normalized on a 0-10 scale (* representing 10).

Dockres can characterize an ensemble of poses in several ways. (1) A histogram can be prepared to show distribution of a number of poses found to be closest to each residue in the macromolecule - the distance between a ligand and a residue is obtained as the shortest distance between any ligand-residue atom pair. One example is shown in Figure 2, which illustrates the distribution for residues 101-150 of the protein uPAR/α5β1 [6]. (2) For each residue distribution of scores of the poses that are closest to it is also calculated. Figure 3 shows the score distribution plot corresponding to the same set of resides depicted in Figure 2. (3) Optionally, one can print RMSDs between different poses of the same ligand (if any) in the top-scoring list. Finally, (4) the overall number of poses with scores falling in 1 kcal/mol bins (starting from the highest score) is printed, which may be helpful to decide on what number of top-scoring poses to extract.

Once the list of top-scoring poses is printed, a new list can be requested where the poses included are limited according to a set of criteria specified by a user. The following criteria are implemented: (1) limit to poses to those that are closest to one of specified residues; (2) limit to the poses that are within a user-specified threshold from a selected macromolecule atom (presumed to be a representative of the binding site); (3) limit the ligands listed to those whose formal charge is between a minimum and maximum as specified by a user; and (4) limit the ligands listed to those whose molecular weight is below a maximum selected by the user. When a binding site is specified, the user has the option to limit the ligand-residue distance calculation to a particular type of atom (instead of any ligand atom) nearest to the residue. The filtering can be repeated with different threshold values. Filtering by a different binding site atom, however, requires a new run of Dockres.

**Figure 4:**
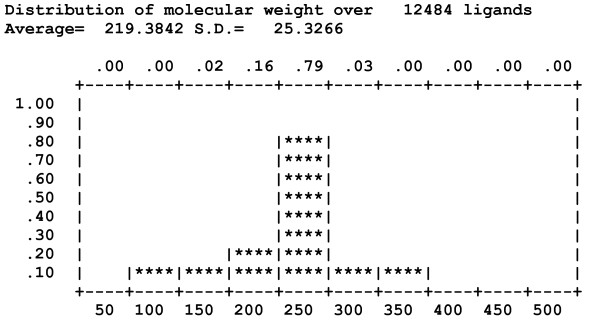
**Distribution of the number molecular weights in the library used to screen uPAR/α5β1**.

In addition to sorting and filtering the docked poses, Dockres also provides detailed statistics on the ligand set (library) used in the screening. Currently this statistics includes the distribution of a number of molecular properties in the ligand set: molecular weight; formal charge; number of rotatable bonds; number of rings; number of hydrogen-bond donors; number of hydrogen-bond acceptors; number of NO_2 _groups; and for each chemical element occurring in the ligand set the number of such atoms. Figure 4 shows the distribution of the molecular weights in the library used in two recent studies [6,7]

Details of the input, output and file formats are described in hyperlinked documentation, which is part of the distribution and can be accessed at http://inka.mssm.edu/~mezei/dockres.

### Files required by the program

The following files (the notation **macro **stands for the name of the macromolecule file's name without **.pdbqs **or **.pdbqt **extension) are required by Dockres: (1) the macromolecule file (used as the target of docking); (2) for Version 4 runs optionally a file with the flexible part of the target with **pdbqt **extension (default name: **macro_flex.pdbqt**); (3) the Autdock grid-parameter file (the one with the **.gpf **extension, used as the input to the Autogrid run); (4) the Autdock docking result files (the ones with the **.dlg **extension); and (5) a file called **macro.dir **that the user has to prepare prior to running Dockres (e.g., by running the script **getdir.csh **- see the Appendix) containing the path to the grid-parameter file and the paths to the Autdock docking result files **(.dlg**).

### Files created by the program

Dockres will create the following files: (1) a file called **macro.res **where all results are printed (if it is already present, it will write instead to **macro_N.res **where N is the smallest integer such that no file with that number exists); (2) a file called **macro.ckp **containing all the information gathered from the input files (checkpoint file), allowing the repeated extraction of data with different filtering criteria without having to perform the time-consuming scan of the **.dlg **files; and (3) optionally, PDB file(s) containing extracted ligand poses with the macromolecule.

The file **macro.res **will contain (1) the description of the input and filtering parameters; (2) optionally, information about each docked pose; (3) the list of top-scoring ligand poses; and (4) the various statistics over the ligand database and the docked poses described above.

For each top-scoring pose, Dockres lists the (1) energy or free energy score; (2) the number of times this pose was found (*m *in Eq. 1 above); (3) ligand identifier as deduced from the file name read from the file **macro.dir **and ligand sequence number in the file **macro.dir**; (4) ligand molecular weight; (5) number of hydrogen-bond donors, acceptors, rotatable bonds, rings and NO_2 _groups in the ligand; (6) total charge of the ligand; (7) the macromolecule atom (index, name, residue number) nearest to the ligand; (8) all other contacts between the ligand and the target (defined as pairs of atoms on the ligand and the target that are mutually closest to each other) and (9) the chemical formula of the ligand.

### Running the program

Dockres is run interactively. The program will ask the user the names of the files required and the various parameters defining the extraction, and listing of results. Once the input information is given, the **.dlg **files are read and the coordinates and scores are extracted from each. This may take some time - for larger libraries the program periodically will print a report of the progress. Once the data is gathered, a checkpoint file is written and the sorting and filtering starts.

First, Dockres prints on the terminal the list of the top-scoring poses and a plot showing the distribution of the location of poses over the macromolecule's residues. Next, the user is given the option to (a) extract docked poses; (2) generate the same distribution restricted to a set of ligands specified by the user; (3) repeat the extraction of statistics subject to the criteria discussed above. Such repetitions are instantaneous, since the program does not have to read the docking output files again for this function.

When requested, the poses extracted are written on a PDB file containing both the macromolecule and the selected poses. The default selection is the list printed, but the user can specify the list of poses to extract. For flexible macromolecule, each pose will result in a complete file with the macromolecule and the ligand whose name will be a combination of the name of the macromolecule, the ligand and the pose number. For rigid macromolecule it is also possible to generate a single file with the different poses added to the macromolecule as additional residues.

### Support utilities

A full list of the utilities provided in the distribution is given in the Appendix. In this section, the functionalities of the major utilities are described. Foremost among the utilities is the script **fullscreen.csh **that performs the screening of the library on a user-specified number of processors. It reads from the terminal all the parameters of the docking and keeps submitting docking runs until the specified number of processors is reached. The number of processors can be changed by the user while the script is running. There is also an option to have the script check for jobs in waiting state and adjust the number of CPUs used to adapt to changing load on the system. The library is assumed to be in Tripos' **.mol2 **format or in Autodock's **.pdbqt **format, in individual files. The user also has to enter the directory paths to the Autodock executables, to the Python executable (pythonsh) and to the Python utility library (pythonutil) as well as the maximum number version of the script runs on systems with the Sun grid-engine (URL: http://gridengine.sunsource.net/) as the queuing system, with systems running the Launcher utility developed at the TACC (URL: http://www.tacc.utexas.edu/) as well as on generic Unix/Linux systems in a single-CPU mode. Different queuing systems require extension of the scripts **fullscreen.csh **and **screenlist_loop_4.csh **and may require the creation of a version of the **dockit_gridengine.csh **corresponding to the new system. Currently work is under way to extend the scripts to run using the Condor queuing system http://www.cs.wisc.edu/condor/.

Screenings done on different conformations of a macromolecule (e.g., different structures from an NMR ensemble) can be concatenated by the program **compare_pose.f**. This program combines the top-scoring poses from all screening runs of each ligand in the vicinity of each residue and sorts this list by their combined scores.

**Figure 5:**
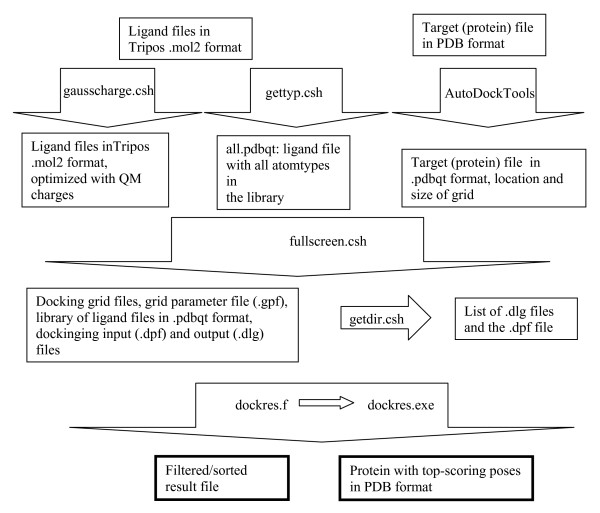
**Flow chart describing the various steps involved in virtual screening using Dockres and its supporting utilities**.

Figure 5 presents a flow chart of the various processes involved in virtual screening and the role of each program or script described in this paper.

## Results

Dockres has been used for the processing of virtual screening of a library of about 13,000 small molecules in recent studies. Some of the hits have been experimentally verified [6,7]. The feature showing the environment of the docked poses was found to be helpful in identifying selective lead compounds [7]. In this study our virtual screening identified inhibitors of the endothelin-converting enzyme-2 (ECE-2), a member of M13 family of zinc metallopeptidases. These compounds were also found to inhibit ECE-1, a close analogue of ECE-2. By examining the binding poses of these ligands we searched for commercially available analogues [8] that would exploit the difference between the binding sites of the two proteins for selective binding to ECE-2 only. Two such compounds were found, exhibiting 5-6 times weaker inhibition than the first set.

## Conclusions

Dockres is available at the URL http://inka.mssm.edu/~mezei/dockres. The distribution includes the source code, tested under Linux and a variety of UNIXes, the documentation, and the utility programs and scripts as listed in the Appendix.

## Competing interests

The authors declare that they have no competing interests.

## Authors' contributions

MM developed the algorithms and wrote the software. M-MZ contributed to the design of the system and its experimental tests. All authors have read and approved the final manuscript.

## Appendix

### List of utilities provided by the Dockres distribution

**splitmol.f **(written by D.A. Gschwend and adapted to generic Fortran by M. Mezei) reads in a file containing several **.mol2 **or pdb* structures and creates several files with a limited number (e.g., one) of structures in each.

**filtermol2.csh **filters a library of .mol2 files: drops files whose molecular weight exceeds an input limit and, for files whose name is of the form **x.y1.mol2**, **x.y2.mol2**, ... keeps only **x.y1.mol2**. It uses the program **filtermol2.f **to check the molecular weight.

**get_typlist.csh **reads all the ligand structures in a directory, extracts a list of Autodock 4 atom types and creates a file **all.pdbqt **with atoms having all the atomtypes found - this file will be used to generate all the grids needed by Autodock.

**mol2togauss.f **prepares an input file for Gaussian [9] to generate partial charges and, optionally, to run a geometry optimization.

**gausstomol2.f **extracts the charges and coordinates from the Gaussian output file and replaces the values in the **.mol2 **file.

**gausscharge.csh **runs **mol2togauss **and **gauss2mol2 **for a whole library. It uses an additional script **mol2togauss.csh **and the program **prepmol2.f**.

**fullscreen.csh**, described above, runs the scripts **screenlist_loop_3 **or **screenlist_loop_4 **for Autodock 3 or 4, resp. These, in turn, run the executable **prepmol2 **and a system-dependent script dockit_*.csh. The current version has the capability to run multi-CPU jobs on systems running the Sun grid engine under Linux and under OSX, for the TACC Ranger systems running the Launcher utility developed there, for generic Unix/Linux shared-memory systems as well as the capability of running on any generic Unix/Linux system in single-CPU mode.

**prepmol2.f **checks a **.mol2 **file to make sure that it does contain charges, the charge sum is integral, and it represents a single molecule.

**getdir.csh **looks into the directory of docking log files and prepares the file **macro.dir **specifying the name of the grid parameter file and of all the logfiles from the different docking runs.

**compare_pose **reads the **.res **files created by **dockres **from docking of the same ligand library to different conformations of the same macromolecule and combines the results as described above.

**clean_dock_dir.csh **removes all files with extension **mol2**, **new**, **pdbq**, **pdbqt**, or **dpf**. These are the extensions of files that the screening script **fullscreen.csh **creates.

**compressdir.csh**: compresses all files in a directory.

**uncompressdir.csh**: uncompresses all compressed files in a directory.

**clean_dock_dir.csh**,**compressdir.csh**, and **uncompressdir.csh **have been written to get around the known problem that C-shell is unable to deal with a long list of files specified with the wild-card symbol *.

## References

[B1] Schneider G Böhm H-J Virtual screening and fast automated docking methods Drug Discovery Today 2002 7 64 70 11790605 10.1016/s1359-6446(01)02091-8

[B2] Klebe G Virtual ligand screening: strategies, perspectives and limitations Drug Discovery Today 2006 11 580 594 10.1016/j.drudis.2006.05.012 16793526 PMC7108249

[B3] Kirchmair J Markt P Distinto S Wolber G Langer T Evaluation of the performance of 3D virtual screening protocols: RMSD comparisons, enrichment assessments, and decoy selection--What can we learn from earlier mistakes? J Comput Aided Mol Des 2008 22 213 228 10.1007/s10822-007-9163-6 18196462

[B4] Morris GM Goodsell DS Halliday RS Huey R Hart WE Belew RK Olson AJ Automated Docking Using a Lamarckian Genetic Algorithm and an Empirical Binding Free Energy Function J Comput Chem 1998 19 1639 1662 10.1002/(SICI)1096-987X(19981115)19:14<1639::AID-JCC10>3.0.CO;2-B

[B5] Ruvinsky AM Role of binding entropy in the refinement of protein-ligand docking predictions: analysis based on the use of 11 scoring functions J Comput Chem 2007 28 1364 1372 10.1002/jcc.20580 17342720

[B6] Chaurasia P Mezei M Zhou M-M Ossowski L Computer aided identification of small molecules disrupting uPAR/a5B1 - integrin interaction; a new paradigm for metastasis prevention PloS ONE 2009 5 9 1487 1499 10.1371/journal.pone.0004617 PMC2643475 19242538

[B7] Gagnidze K Sachchidanand Rozenfeld R Mezei M Zhou M-M Devi LA Homology modeling and site-directed mutagenesis to identify selective inhibitors of endothelin-converting enzyme-2 J Med Chem 2008 51 3378 3387 10.1021/jm7015478 18507370 PMC2706147

[B8] Irwin JJ Shoichet BK ZINC - A Free Database of Commercially Available Compounds for Virtual Screening J Chem Inf Model 2005 45 177 182 10.1021/ci049714+ 15667143 PMC1360656

[B9] Frisch MJ Trucks GW Schlegel HB Scuseria GE Robb MA Cheeseman JR JA Montgomery J Vreven T Kudin KN Burant JC Gaussian-03 2004

